# Factors predicting cosmesis, late effects (LEs) and patient reported measures (PROMs) in high-risk breast cancer (BC) treated with hypo-fractionated whole breast radiotherapy (HF-WBI): real world outcomes from a developing country

**DOI:** 10.3332/ecancer.2025.1877

**Published:** 2025-03-20

**Authors:** Chandrashekhar Mishra, Sushma Agrawal, Punita Lal, Gyan Chand, Gaurav Agarwal

**Affiliations:** Department of Radiotherapy, Endocrine Surgery, Sanjay Gandhi Post Graduate Institute of Medical Sciences, Lucknow 226014, India; a https://orcid.org/0000-0002-7260-6509

**Keywords:** large operable, locally advanced breast cancer, brest conservation, cosmesis, late effects, patient reported outcome measures

## Abstract

**Background:**

Breast cancer (BC) in low–middle income country commonly presents as large operable/locally advanced BC (LO/LABC). Advances in treatment have gradually increased the probability of breast conservation in this population. Hypo-fractionated whole breast radiotherapy (HF-WBI) has become the standard of care. Literature on cosmesis in high-risk BC after HF-WBI is sparse. Hence, we evaluated the cosmesis, late effects (LE) and patient-reported outcome measures (PROM) and factors affecting it in women with high-risk BC.

**Materials and methods:**

Patients who underwent upfront breast-conserving surgery (BCS) or after neoadjuvant chemotherapy underwent physical cosmetic assessment (CA), LEs evaluation and PROM using European Organisation for Research and Treatment of Cancer, Radiation Therapy Oncology Group and BR23 questionnaire, respectively. Patient, tumour and treatment-related factors were correlated with CA, LE and PROM by univariate and multivariate analysis.

**Results:**

186 women underwent BCS, but only 82 could participate due to COVID-19 pandemic. Prevalence of high-risk features were: >3 cm tumour in 37%, 50% node positive, 100% received chemotherapy (CT), grade 3 in 57% and Her-2 (enriched)/TNBC in 70%. At a median follow-up of 5 years, we found 56% of patients had adverse global cosmesis. Factors responsible for adverse cosmesis were tumour size [>3 cm, HR 2.3], node positivity [HR 0.3], receipt of CT [HR 6.5], large resection volumes [HR 4.6], large breast volume [HR 1] and supraclavicular radiotherapy in 43% [HR 0.7]. Dosimetric factors found significant for adverse cosmesis were breast volume receiving 107% dose (> than 10 cubic centimetres (cc), HR 5) and breast volume receiving 100% dose (> than 120 cc, HR 5). The incidence of arm oedema was 6%, shoulder stiffness 20%, brachial plexopathy 4% and factors significant for LE were tumour size > 3cm [HR 9], breast volume receiving 107% [HR 6] and co-morbidity [HR 3]. PROM revealed that 69% had poor body image, 35% had breast pain and 47% were sexually active. Arm score had a correlation with positive nodal status (HR 4.3), and breast score with large breast volume (HR 5.4) and premenopausal status (HR 7.3).

**Conclusion:**

Our data reveals that 75% of our population have large breast, the presence of high-risk features in 50% women, which resulted in a high incidence of adverse global cosmesis, (56%), LE and PROM (poor body image, breast pain and low sexually activity).

## Introduction

Breast-conserving surgery (BCS) is the standard of care in early breast cancer (BC) and large operable (LO) disease adequately down-staged with chemotherapy (CT) which is followed by whole breast radiotherapy (RT) and boost to the resection cavity if indicated [1]. Results from large randomized trials have confirmed that hypo-fractionated whole-breast irradiation (HF-WBI) is safe and efficacious in comparison to conventional fractionation in early-stage BC [2, 3]. Moreover, the update of the American Society of Radiation Oncology evidence-based guidelines on radiation therapy for the whole breast confirmed that hypo-fractionated whole breast radiotherapy (HF-WBI) represents the preferred option for patients with early-stage BC, regardless of age, CT administration and breast size and recommends minimization of breast volume receiving >105% of the prescription dose [1]. After this publication, HF-WBI has been widely adopted due to its efficacy and logistics advantage. Real world data have confirmed the safety and efficacy of hypo-fractionated RT in early BC and locally advanced BC in various studies [4, 5]. Cosmesis is an important endpoint of the successful implementation of breast conservation. While the reported cosmesis with HF-WBI is good in early BC, there is sparse literature on the cosmesis and late effects (LEs) in with large operable/locally advanced BC (LO/LABC).

In our country, women more often present with LO/LABC [6]. Though we have reported the safety and efficacy of HF-WBI in LO/LABC, cosmetic outcome and LEs analysis have not yet been conducted [5]. Hence, we conducted a one-time cross-sectional study of women who underwent BCS with at least 3 years follow-up (after which cosmesis usually stabilises) to ascertain the cosmetic assessment (CA) and LE in the arm and shoulder and patient-reported outcome measures (PROM).

## Materials and methods

This was a prospective observational and cross-sectional single-centre study in a regional cancer centre setting in low–middle income country during the COVID-19 pandemic. After approval of the institute ethics committee (2018-82-IP-EXP), all patients of BC who underwent BCS during the period 2013 to 2017, who had completed 3 years after treatment and could attend the clinic for cosmesis and LE assessment for arm and shoulder were enrolled in this study. Patients were telephonically called up to attend Out Patient Department (OPD) over a 6-month period. Women underwent upfront BCS in those who were suitable, while LO and locally advanced tumours underwent BCS after adequate downstaging with standard neoadjuvant chemotherapy (NACT). The institutional protocol of NACT comprised of 3 weekly 4 cycles of Adriamycin cyclophosphamide followed by 4 (3 weekly) or 12 weekly taxanes. This is followed by BCS (wherever possible) and axillary lymph-node dissection (ALND). Thereafter all were treated with HF-WBI whole breast RT and supraclavicular RT (in histopathological node-positive patients) by 3DCRT technique to a dose of 40 Gy/15 fractions in 3 weeks [7]. This was followed by a boost (10–12 Gy/4–6 fractions) to tumour bed in all patients. intensity modulated radiation therapy (IMRT) (field-in-field technique) was used when necessary to improve the homogeneity of target volume coverage. All hormone receptor-positive patients received either tamoxifen or letrozole according to their menopausal status and trastuzumab was administered to Her-2 neu positive women who could afford it.

### RT technique

Patients were imaged in the supine position using a recommended breast board for immobilisation. Reproducibility of position was achieved by matching orthogonal laser beams with anterior and bilateral skin tattoos. The clinical target volume (CTV) was defined to include soft tissues of the whole breast down to the deep fascia, but not including underlying muscle and ribcage, nor overlying skin and excision scar. The inferior, medial and lateral limits of the breast were localised by inspection and palpation of the breast before CT images were taken. The superior border was defined in relation to the suprasternal notch. The planning target volume (PTV) for the whole breast included these limits to the CTV plus a 1 cm expansion in four radial directions. The RT plan for all patients was generated using two standard tangential fields with non-divergent posterior beam edges with a single isocentre technique. The recommended maximum lung distance and maximum heart distance in the treatment volume were <2 and <1 cm, respectively, using cardiac shielding, where appropriate, was introduced using a multi-leaf collimator (MLC) or other shielding technique. The prescription point was halfway between the anterior lung surface and the skin surface on the perpendicular bisector of the posterior treatment beam edge. If a standard wedged pair was insufficient, full dose compensation was performed using intensity-modulated fields generated by forward planning. In practice, a forward-planned MLC segment method was the dose compensation technique. Standard treatment fields were applied, and dose homogeneity was evaluated. Any hot spots of dose greater than 107% were minimised.

### Cosmesis assessment

Two physicians (SA and CM) together performed a one-time physical cosmesis assessment of the ipsilateral breast (CA) and compared it with the contralateral breast using the European Organisation for Research and Treatment of Cancer (EORTC) scoring system [8]. This system categorises the appearance of the surgical scar; breast size; breast shape; nipple position; skin colour and shape of areola by comparing the treated breast with the untreated breast into a 4-point scale. The results are classified into one of the following categories: ‘0’ represents an excellent result; ‘1’ a good result; ‘2’ a fair result and ‘3’ a poor result. Additionally, a global cosmetic score was also tabulated by averaging the scores of all the above criteria and categorising them into the above 4 categories: ‘0’ represents an excellent result; ‘1’ a good result; ‘2’ a fair result and ‘3’ a poor result.

### LEs assessment

LEsin the arm and shoulder (LE) were assessed using the Radiation Therapy Oncology Group scoring system. [9] This system evaluates the pigmentation /atrophy/ulceration of the skin of the arm and shoulder, arm and shoulder pain, arm oedema and brachial plexopathy (if symptoms of pain, paraesthesia, numbness or other sensory symptoms are present). This system categorises the various changes into four grades ranging from no changes (grade 0) to maximum change (grade 3). Arm oedema was categorised into various grades (with the normal arm as comparator) as follows: Grade 1: 5%–10% inter-limb discrepancy in volume or circumference at point of greatest visible difference; swelling or obscuration of anatomic architecture on close inspection; pitting oedema. Grade 2: 10%–30% inter-limb discrepancy in volume or circumference at point of greatest visible difference; swelling or obscuration of anatomic architecture on close inspection. Grade 3: >30% inter-limb discrepancy in volume or circumference at point of greatest visible difference; swelling or obscuration of anatomic architecture on close inspection, interference with active daily living. Grade 4: progression to malignancy.

*PROMs were collected with the EORTC-BR23 questionnaire in the local language (validated in Hindi).*

Additionally, demographic data, treatment treatment-related data (pertaining to CT, surgery and RT) were also collected for correlation of these factors with CE, LE and PROM. Apart from RT dose, fields and technique, some additional RT-related dosimetric data were collected and which need explanation are as follows.

### Dose inhomogeneity

Volume of 107% and 100% isodose (A parameter representing the proportion of breast volume receiving >100% of the prescribed dose was created from the dose–volume histogram data. Raw data for absolute breast volume (representing breast size) and volume of breast tissue receiving >100% of the prescribed dose were summarised. The 100% reference point was selected because 107% represented a limited part of the dose–volume distribution. In the patients with any volumes receiving >107%, the median percentage volume of treated breast receiving dose >107% was also noted.

### Baseline breast volume

Assessment of breast volume for the current study was estimated (in cubic centimetres (cc)) using RT planning X-ray CT images, but since the superior, inferior, medial and lateral limits of the CTV were defined by inspection and palpation, a breast CTV was not calculated. Instead, tissue lying within the 50% isodose line was selected as an appropriate surrogate measure for breast volume, which is less dependent than the 95% isodose line on the planning system and on the different X-ray energies.

### Follow-up

After completion of treatment, patients are called every 3 months for the first 2 years for a clinical assessment and, thereafter, 6 monthly up to 5 years. Thereafter follow-up was once a year.

### Data and statistical analysis

The ‘quite a bit’ and ‘very much’ categories of all endpoints of CA, LE and PROM were combined into one category (moderate/marked), similarly none and a little were combined into another category (mild) which resulted in a 2-point scale. Logistic regression was used to assess the effect of all the tumour and treatment variables on the risk of cosmesis, LEs and PROMs. Each factor was first tested alone in a univariate model, and then significant factors were tested in a multivariate analysis to test whether the effects were independent of each other. A value of *p* = 0.05 was taken as significant. Analysis of PROM using BR23 was used as stated in the questionnaire. The raw scores obtained from the questionnaire were linearly transformed to a 0–100 range.

## Results

**Figure 1.**
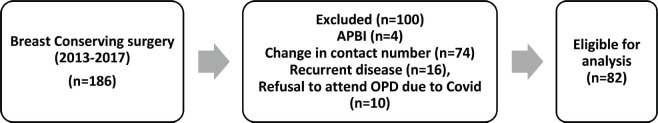
Consort diagram.

Out of 186 patients who underwent BCS followed by HF-WBI (2013–17), only 86 patients could attend OPD for cosmesis and LE assessment. Four among these underwent accelerated partial breast radiation, therefore, excluded from the analysis to ensure data of a homogenously treated population. The causes of non-enrolment of 100 patients in this study were: change in contact number (*n* = 74), recurrent disease (*n* = 16) and refusal to attend clinic due to the pandemic (*n* = 10) (Figure 1). The median age of the cohort was 47.50 years (IQR range,30–76 years), majority of tumours were T2 (69.5%) and node-positive disease (46.3%) (Table 1). Since the median tumour size was 3 cm, 30% of women received NACT followed by BCS. The type of BCS was lumpectomy (80.5%), onco-plasty (14.6%) and revision lumpectomy (4.9%). 69.5% of women underwent axillary dissection and the rest sentinel lymph node biopsy (SLNB). Of all patients who received a boost, 42.7% received supraclavicular radiatotherapy whereas axillary RT was given to only 2.4% of patients (Table 2). Though a large proportion of patients were Her-2 neu positive (44%) only 10% could afford trastuzumab.

The median volume of excision was 100 cc (IQR 60–141 cc, derived from excised specimen volume), the median PTV of boost was 100 cc (IQR 77–163 cc), median breast volume was 1,600 cc (IQR 1,300–2,000 cc), median breast volume receiving 100% dose was 120 cc (IQR 83–213 cc) and median breast volume receiving 107% dose was 10 cc (IQR 0–16 cc). Two categories were created in all the above variables for correlation with CE and LE: (< and > than 100 cc for excision volume and PTV boost), (<1,800 and > than 1,800 cc for breast volume as no meaningful correlation was found with the median value of 1,600 cc), (< and > than 120 cc for breast volume receiving 100% RT dose) and (< and > than 10 cc for 107% dose volume). Women with breast volume <1,800 cc were considered medium size breasts and >1,800 cc as large size.

### Factors predicting cosmesis

**Figure 2.**
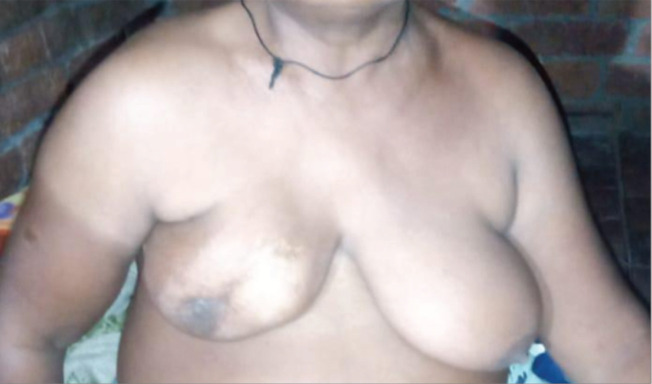
An example of patient with poor cosmesis and marked skin changes.

**Figure 3.**
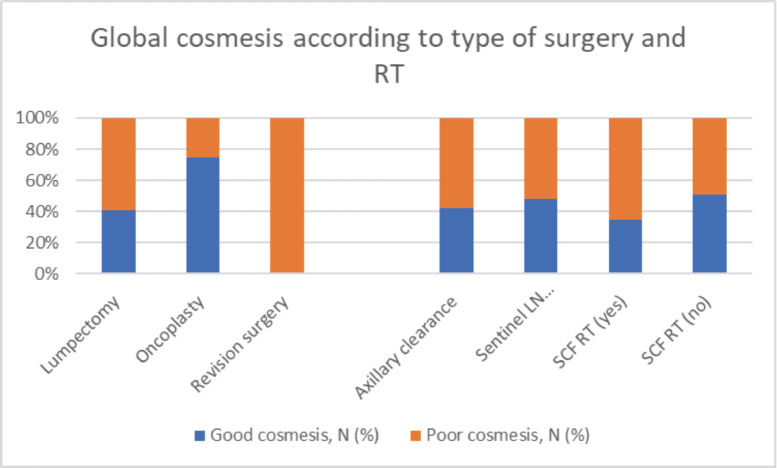
Impact of type of surgery on global cosmesis.

At a median follow-up of 60 months, the global cosmetic score of the entire cohort was poor in 56% of patients (Figure 2). The other ascertained parameters of CA, LE and PROM are listed in Table 3. On univariate analysis, as compared to women with good cosmesis, women with adverse global cosmesis had significantly higher resected volume (>100 cc in 67% versus 27%, *p*-value = 0.000), higher dose heterogeneity (107% volume >10 cc in 28% versus 2.8%, *p*-value = 0.009), large breast volume (70% versus 30%, *p* = 0.08), higher breast volume receiving 100% RT dose (64% versus 36%, *p* = 0.08), likelihood of receiving NACT (81% versus 19%, *p*-value = 0.003) and SCF RT (66% versus 34%, *p* = 0.09) (Table 4, Figures 1–3). On multivariate analysis of factors affecting various domains of cosmesis and global cosmesis, menopausal status (HR 3.4), volume of resection (HR 3.8) and volume of breast receiving 107% dose (HR 11) had a significant impact on breast size. Receipt of NACT (HR 8.9) and volume of breast receiving 107% (HR 17.9) had a significant impact on breast shape. The volume of the breast receiving 107% (HR 17) had a significant impact on the nipple position. Receipt of NACT (HR 4.4) and volume of breast receiving 107% (HR 3.8) had a significant impact on scar appearance. Volume of resection (HR 4.6) and receipt of NACT (HR 4.4) had a significant impact on global cosmetic outcomes (Table 5).

### Factors predicting LEs in arm and shoulder

Assessment of LE by physician revealed skin changes in the breast and shoulder in 12% of patients, brachial plexopathy in 4%, shoulder stiffness in 20% and arm oedema in 6%, while according to PROM shoulder and arm pain was found in 35%, shoulder stiffness in 20% and arm oedema in15%. On multivariate analysis of factors affecting physician-assessed LE in arm and shoulder, tumour size had a trend toward significance (HR 9) for arm oedema, volume of breast receiving 107% had a significant impact on brachial plexopathy (HR 6.1) and shoulder stiffness (HR 4). Presence of comorbidity also affected shoulder stiffness (HR 3.9) (Table 6).

### Factors predicting PROM

**Figure 4.**
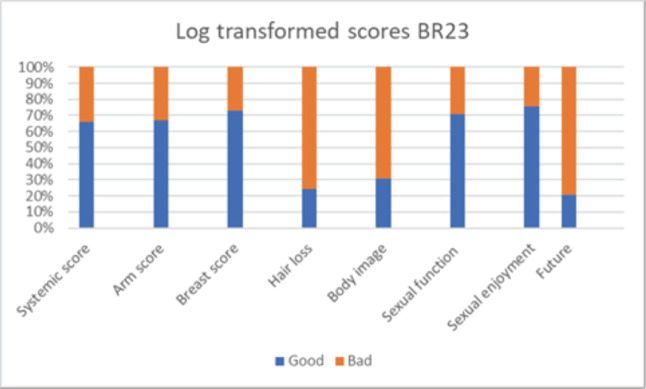
Patient reported outcome scores (BR23).

Patients reported breast pain in 35%, breast oedema in 11%, breast sensitivity in 15% and change in the skin of the breast in 64% (Figure 4). Sexual parameters like sexual interest was present in 46%, 47% were sexually active and sex was enjoyable in 45%. The log-transformed systemic symptom score was poor in 34% of women, arm score was poor in 33%, breast score was poor in 27% and hair loss was bothersome for 75% of women. Body image perception was poor in 69% of women, sexual function and enjoyment were poor in 30% and 25% of women and 80% of them were bothered about their future. On multivariate analysis of factors affecting symptom and functional scales, postmenopausal status was associated with higher arm score, breast score, hair loss and sexual function decline. Node-positive status was also associated with higher arm scores. High breast volume and premenopausal status had a trend toward higher breast scores. T size more than 3 cm and receipt of SCF RT shows significantly higher altered body image (Table 7, Figure 3).

## Discussion

Our study comprised patients with high-risk features like T2 or more [95%], node positive [50%], receipt of CT [97%] and SCF RT [43%] which resulted in adverse cosmesis in 56% of patients, whereas the reported cosmesis in START B in patients with early-stage tumours and node-negative tumours (70% patients were T1, 72% were node negative) resulted in any change in breast appearance (mild or marked) at 5 years recorded using the consensus scores in only 39.3% patients [10]. Our data were comparable to the high incidence of poor cosmesis by physician assessment of 38% with hypofractionation as compared to 44% with conventional fractionation in another study from our country [11]. This incidence of poor cosmesis by physician assessment increased to 43% with HF boost as compared to 50% with conventional fractionation boost. In our series, all patients received HF-boost. Similar observations of poor cosmesis have been observed with hypo-fractionated RT in women with high-risk features in 61% [12]. The EORTC study reported a significant association of the risk of moderate to severe fibrosis with the administration of CT and or tamoxifen in premenopausal or postmenopausal women, respectively [13]. The DBCG group observed excellent to good cosmesis in only 50% of patients in a population of patients with predominantly T1 tumours (80%) treated with conventional fractionation, where apart from physician assessment, nipple position was also ascertained with BCCT.core software [14].

Apart from demographic high-risk features, the surgical factors contributing to the high incidence of adverse cosmesis in our patients was large resection volumes (>100 cc) in approximately 50% of patients which is a reflection of 37% tumours being more than 3 cm. In a study of CA in 598 early BC, the resection of 70 cc or more of breast tissue was more common among the failed patients than among matched patients with good or excellent cosmesis [15]. For safe and cosmetically acceptable breast-conserving therapy in patients with large BCs, the tumour volume should be reduced preoperatively by NACT, and lost tissue volume should be replaced after wide local excision by oncoplastic techniques. During the period of assessment in the index study, only 12 patients with oncoplastic technique turned up for CA and the cosmetic outcome of these patients was good in 75% of patients as compared to good cosmesis in 44% of patients who underwent non-oncoplasty surgery. As more oncoplastic techniques are gradually being adopted by our surgeons, the cosmesis rate is likely to improve in the near future.

The RT-related factors that contributed to poor cosmesis were large breast volume and volume of breast receiving 107% or more dose. In our series, we estimated the surrogate of breast volume (from the treatment planning scan), i.e., the volume encompassed by 50% isodose as mentioned in IMPORT Low [16]. They reported a median volume of 778, 1,114 and 1,357 cc as cut-off volumes for small, medium and large breasts when correlated with cup size. Since our series was an ambispective analysis and we did not have the previous bra cup size of our patients to correlate with breast volumes, we sought to correlate with published data. In our series, the lower 25% cutoff of breast volume was 1,300 cc which matches with cutoff for large breast volume in START B trial. Since 75% of women in our study had large breast volume, this seems to be responsible for the high incidence of adverse cosmesis. Our high incidence of adverse cosmesis can also be explained by the delivery of boost in all patients. Goldsmith *et al* [17] reported a hazard ratio of 1.8 for poor cosmesis in patients receiving 100% dose to more than 42% volume of the breast while we found an HR of 12. Adoption of IMRT techniques or simultaneous integrated boost improves dose in-homogeneity and results in superior overall cosmesis provided the boost volume is small and the EQD2 of boost dose is 60 Gy or less as observed in the IMPORT HIGH trial [18–20]. Nodal positivity mandates RT to SCF with or without axillary RT depending on the risk factors. We observed a higher incidence of adverse cosmesis in women who received RT to SCF (HR 0.7). Similar findings have been reported in a study where 89% of patients treated with a tangent pair technique had excellent results at 5 years when treated with tangential beams alone as compared with 69% of patients treated with tangents and SCF RT [13]. SCF RT has also been found to contribute to poor cosmesis in some studies with hypofractionation [21]. Publication of NSABP-51 results and RAPCHEM allows us to adopt de-escalation of regional nodal RT in responders to NACT, thus resulting in superior cosmesis and LEs as well [22]. The other factors that resulted in poor cosmesis were postmenopausal status (60%) and upper outer quadrant tumours. Though the literature suggests adverse cosmesis in the inner quadrant and central tumours, our series had low numbers of other quadrant tumours (only 25%) [23]. The most significant factors affecting global cosmesis were breast size, shape and nipple position.

The gold standard for cosmesis assessment is clinical assessment and comparison with the normal contralateral breast which allows a 3-D view whereas this is not possible with standard photographs. We did not indulge in complex methods of CA like BCTOS or the use of BCCT software because these cannot be used in the clinic daily even though they are more accurate. Since we investigated women with a median follow-up of 5 years, a one-time cross-sectional study is adequate, as any further change in cosmesis is usually not observed after 5 years of RT completion.

### Late effects

Our findings reveal that tumour size more than 3 cm significantly contributed to arm oedema (6%) and sentinel LN procedure led to a lower (4.3%) incidence of arm oedema as compared to 9% with axillary clearance. The START B trial reported 8% arm oedema, and 18% shoulder stiffness with hypofractionation as compared to 6% and 8% with conventional fractionation. The Danish study of early BC treated with conventional fractionation reported that the number of positive axillary nodes >10 and axillary RT increased the arm oedema rate from 7% to 28%, arm pain from 13% to 28%, shoulder stiffness from 0% to 8% [24]. Their 10-year follow-up study stated that 64% of patients had one or more subjective loco regional symptoms like pain, swelling of the arm and decreased shoulder mobility. In our series, a higher incidence of shoulder stiffness was observed in those who received SCF RT (26% versus 18% in those who did not) which is comparable to the literature. Factors significantly impacting shoulder stiffness were the presence of comorbidity and the volume of breasts receiving a 107% dose. Adoption of targeted axillary sampling rather than ALND in patients receiving NACT is likely to decrease the incidence of arm oedema [25] De-escalation of RT to SCF in complete nodal pathological response is likely to decrease the incidence of LEs in the arm and shoulder.

### Patient-reported outcome measures

Patient-reported cosmetic outcomes and satisfaction have become critical endpoints of BCT. Our data reveals that the breast score was poor in 26% of women in the index study, and their perception of body image was poor in 69%. There is enough data which states that women who were dissatisfied with their cosmetic results had an impaired body image. Data from START B trial reported a 20% incidence of patient reported poor cosmesis with two field hypo-fractionated breast RT in early BC [3]. Breast score was poor in women with breast volume (>1,600 cc) (HR 5.4) and premenopausal status (HR 7.3) which has been reported in the literature. Data from breast IMRT study also revealed that breast volume, young age and postoperative infection were independent risk factors for breast score. Breast pain affects quality of life in survivors and was present in 33% of women in the index study which was similar to START trial. Similarly, 40% of women had marked symptoms in arm and shoulder while the incidence of same was 20% in START trials [10, 24]. In the index study, arm score had correlation with positive nodal status (HR 4.3), which has been reported in literature [14]. In view of the high incidence of breast and arm score, the adoption of oncoplasty techniques in women with HRF is likely to overcome this challenge. Based on the results of de-escalation studies, perhaps avoidance of boost and de-escalation of regional nodal irradiation in complete pathologic responders to NACT is likely to improve cosmetic and LE results [22].

Sexual dysfunction is common in BC survivors and has been found to be associated with feelings of emotional separation in couples, fear of sexual intercourse, lower emotional functioning, poorer body image or co-morbidities [24, 26]. In our series, only 70% of women were sexually active or found it enjoyable, and this was more adversely affected in premenopausal women. This finding is concerning for premenopausal women [27]. Given the fact that BC is aggressive in young women assessment of de-escalation of RT has to be weighed with its safety, although onco-plasty and targeted axillary dissection has proven its efficacy.

The START and IMPORT trial results reveal that PROMs could potentially replace either CA or photographs to assess cosmesis since patients rate their subjective satisfaction with an experience of a range of breast changes, whilst clinicians seek objective adverse treatment effects. Since CA are still widely used all over the globe, an alternative viewpoint is that both PROMs and CAs are necessary as they measure differing aspects of disease experience and are complementary.

## Limitations

The main limitation of our study was only 46% attendance of BCS patients for cosmesis evaluation because of various reasons mentioned earlier. Another limitation was the lack of a baseline picture of the involved breast prior to RT as cosmesis assessment was not planned prior to RT. The other limitation of our study was that the BR 23 questionnaire does not directly evaluate patient-reported cosmesis, but rather variables assessing change in skin appearance, firmness in the area of the affected breast, asymmetry and distortion and shrinkage of the breast as used for START trial would have captured adequate information.

## Conclusion

Our data reveals that 75% of our population have large breast, the presence of high-risk features in 50% women, which resulted in a high incidence of adverse global cosmesis, (56%), LE and PROM (poor body image, breast pain and low sexually activity).

## Statements and declarations

The authors declare that no funds, grants or other support were received during the preparation of this manuscript.

The authors affirm that human research participants provided informed consent for the publication of the images in Figure 1.

All authors contributed to the study’s conception and design. Material preparation, data collection and analysis were performed by (Chandrashekhar Mishra), (Sushma Agrawal). The first draft of the manuscript was written by (Sushma Agrawal) and all authors commented on previous versions of the manuscript. All authors read and approved the final manuscript.

## Data availability

The datasets generated during and/or analysed during the current study are not publicly available due to Institute policy but are available from the corresponding author on reasonable request.

## Conflicts of interest

The authors declare that they have no competing interests.

## Funding

Non-funded study.

## Ethics approval

This study was performed in line with the principles of the Declaration of Helsinki. Approval was granted by the Institute Ethics Committee of University B (Date 2.9.20./No2018-82-IP-EXP).

## Consent to participate

Informed consent was obtained from all individual participants included in the study.

## Figures and Tables

**Table 1.** Baseline demographic characteristics of the cohort.

Parameters	*N* (%)
Age (median and IQ range in years)	47.50 (30–76)
Comorbidity HT/DM	17 (20.7)
Right side Left side	37 (45.1) 45 (54.9)
Premenopausal Postmenopausal	26 (31.7) 56 (68.3)
Quadrant Upper outer Lower outer Rest	62 (75.6) 12 (14.6) 8 (8.5)
T clinical T1 T2 T3 T4 Missing *N* clinical N0 N1 N2 Missing	2 (2.4) 57 (69.5) 18 (22.0) 3 (3.7) 2 (2.4) 44 (53.7) 25 (30.5) 9 (11) 4 (4.9)
Grade: G1/G2/G3	5 (6.1)/30 (36.6)/47 (57.3)
Intrinsic subtype Luminal A Luminal B Her2 type TNBC	5 (6) 18 (22) 36 (44) 23 (28)

**Table 2.** Intervention received by the cohort.

Parameters	*N* (%)
NACT (Yes) NACT (No) Trastuzumab	25 (30.5) 57 (69.5) 8 (9.8%)
Surgery of primary Lumpectomy Onco-plasty Revision lumpectomy	66 (80.5) 12(14.6) 4 (4.9)
Axillary surgery Axillary dissection SLNB	57 (69.5) 25 (30.5)
Vol. of excision <100 cc >100 cc	41 (50) 41 (50)
Hormone therapy Tamoxifen/Letrozole No hormone	13 (15.9%) /23 (28.0%) 46 (56.1%)
Axillary RT given Axillary RT not given SCF RT given SCF RT not given	2 (2.4) 80 (97.6) 35 (42.7) 47 (57.3)
PTV boost vol (IQR 77–163 cc) <100 cc >100 cc	29 (36.7) 53 (65.3)
Breast volume (IQR 1,300–2,000 cc) Medium (includes small) [<1,600 cc] Large (>1,600 cc]	42 (51.2) 40 (48.8)
Volume of breast receiving 100% dose (IQR 83–213 cc) <120 cc >120 cc	43 (52.4%) 39 (47.6%)
Volume of breast receiving 107 % dose (IQR 0–16 cc) <10 cc >10 cc	67 (81.7) 15 (18.3)

**Table 3.** Incidence of cosmesis and LEs as assessed by patient and physician.

Patient assessed variables (BR23)	Present n (%)	Physician assessed variables	Grade 1,2 *n* (%)	Grade 3,4 *n* (%)
Breast pain	29 (35)	Breast size	50 (60)	33 (40)
Breast edema	09 (11)	Breast shape	57 (70)	25 (30)
Breast sensitivity	12 (15)	Nipple position	66 (80)	16 (20)
Skin of breast	52 (64)	Shape of nipple and areola	66 (80)	16 (20)
Altered Body image	56 (69)	Skin on breast	66 (80)	16 (20)
Interested in sex	38 (46)	Scar appearance	50 (60)	33 (40)
Sexually active	39 (47)	Global cosmetic score	36 (44)	46 (56)
Sex enjoyable	37 (45)	Skin of breast and shoulder	10 (12%)	-
Arm edema	12 (15%)	Arm edema	6 (7.4%)	-
Shoulder stiffness	16 (20%)	Brachial plexopathy	8 (10%)	-
Pain shoulder	29 (35%)	Shoulder stiffness	16 (20%)	-

**Table 4.** Univariate analysis of variables affecting cosmesis.

Variables	Good cosmesis % (*n*)	Poor cosmesis % (*n*)	*p*-value
T1, 2 (*n* = 61) T3, 4 (*n* = 21)	49 (30) 28 (6)	51 (31) 72 (15)	0.09
Resected volume <100 cc (*n* = 41) >100 cc (*n* = 41)	73 (26) 27 (10)	33 (15) 67 (31)	0.000
Type of breast surgery Lumpectomy (*n* = 66) Oncoplasty (*n* = 12) Revision surgery (*n* = 4)	**27 (40.9)** **9 (75)** **-**	**39 (59.1)** **3 (25)** **4 (100)**	0.01
NACT (yes) *n* = 25 NACT (no) *n* = 57	19 (5) 54 (31)	81 (20) 46 (26)	0.003
Breast volume Medium (*n* = 48) Large (*n* = 34)	71 (25) 30 (10)	53 (23) 70 (24)	0.08
Breast volume receiving 107% dose <10 cc, *n* = 67 >10 cc, *n* = 15	**34** **2.8 (2)**	33 (72) 28 (13)	0.009
Br. volume receiving 100% dose <120 cc (*n* = 43) >120 cc (*n* = 39)	**49 (21)** **36 (14)**	51 (22) 64 (25)	0.08
SCF RT (yes) (*n* = 35) SCF RT (No) (*n* = 47)	**66 (24)** **34 (12)**	50 (23) 50 (23)	0.09

The bold values in tables are statistically significant which was a p-value of <0.05

**Table 5.** Logistic regression of significant/near significant variables affecting sub-domains of cosmesis and global cosmesis.

Variables	Breast size *p*-value, HR (CI)	Breast shape *p*-value, HR (CI)	Skin breast *p*-value, HR (CI)	Nipple position *p*-value, HR (CI)	Scar appearance *p*-value, HR (CI)	Global cosmetic score *p*-value, HR (CI)
Premenopausal (R) *n* = 26 Post-meno, *n* = 56	0**.08 (3.4)** **(0.8–14)**	0.3 (2) (0.4**–**10)	0.9 (0) (0)	0.1 (10) (0.5**–**180)	0.3 (1.4) (0.4**–**5)	0.3 (0.5) (0.4**–**1.5)
Node negative (R), *n* = 44 Node positive , *n* = 38	0.8 (1.4) (0.1**–**4.9)	0.5 (0.5) (0.05**–**4.8)	1 (0.1) (0)	0.2 (4.6) (0.01**–**3.8)	0.3 (0.5) (0.1**–**2)	**0.06( 0.3)** **(0.09–1.1)**
Resection vol <100 cc (R), *n* = 41 >100 cc, *n* = 41	**0.06 (3.8)** **(0.9–16)**	0.3 (2) (0.4**–**10)	1 (0.1) (0)	0.4 (3) (0.2**–**55)	0.4 (1.3) (0.2**–**5)	**0.005 (4.6)** **(1.6–13.6)**
NACT (yes) *n* = 25 NACT (no, R), *n* = 57	0.3 (2) (0.4**–**10)	**0.006 (8.2)** **(1.8–36.6)**	0.9 (0) (0)	0.3 (3) (0.2**–**41)	**0.009 (4.4)** **(1.4–13)**	**0.009 (6.5)** **(1.6–26)**
Br. volume 107% dose <10 cc, *n* = 67 >10 cc, *n* = 15	**0.004 (11)** **(2–56)**	**0.001 (18)** **(3–100)**	0.19 (6.7) (0.38**–**118)	**0.003 (17)** **(3–109)**	**0.06 (3.8)** **(0.9–16)**	0.1 (5) (0.5**–**48)
Br. volume 100% dose <120 cc, *n* = 43 >120 cc, *n* = 39	**0.9 (1.1)** **(0.09–12)**	0.8 (0.7) (0.06**–**9.3)	1 (0) (0)	0.9 (0) (0)	0.6 (0.3) (0**–**4.3)	0.6 (5) (0**–**49)
SCF RT (yes) *n* = 35 SCF RT (No, R), *n* = 47	**0.06 (0.28)** **(0.07–1)**	0.4 (0.5) (0.09**–**2.9)	0.9 (0) (0)	0.1 (0.1) (0.01**–**0.15)	0.1 (0.4) (0.1**–**1.3)	0.5 (0.7) (0.1**–**1.3)

Statistically significant which was a p-value of <0.05

**Table 6.** Logistic regression of significant /near significant variables affecting LEs in arm and shoulder.

Variables	Arm edema *p*-value, HR (CI)	Brachial plexopathy *p*-value, HR (CI)	Shoulder stiffness, *p*-value, HR (CI)	Skin arm, shoulder *p*-value, HR (CI)
Comorbidity (no, R) (*n* = 65) Comorbidity (yes) (*n* = 17)	0.3 (0.2) (0.02–1)	**0.06 (0.2)** **(0.04**–**1)**	**0.07 (4.8)** **(0.6**–**8.7)**	0.13 (3) (0.7–13)
T <3 cm (R) (*n* = 59) T>3 cm, (*n* = 23)	**0.09 (7.2)** **(0.6**–**76**)	0.2(0.3) (0.4–3.7)	0.6 (1.3) (0.3–5.7)	0.4 (2.2) (0.3–16)
Volume receiving 107% <10 cc (*n* = 67) >10 cc (*n* = 15)	0.9 (0) (0)	**0.03 (6.1)** **(1.1–33.5)**	0**.07 (4)** **(0.8–18)**	0.2 (2.8) (0.4–18)

Statistically significant which was a p-value of <0.05

**Table 7.** Logistic regression of significant/near significant variables affecting PROM.

	Symptom scale (*p* value, (HR))				Functional scale (*p* value, (HR))			
Variables	Systemic score 21 ± 14.6 (0–85)	Arm score 15 ± 21 (0–77)	Breast score 15 ± 12 (0–66)	Hair loss 31 ± 24 (0–100)	Body image 80 ± 22 (16–100)	Sexual function 75 ± 27	Sexual enjoyment	Future 66 ± 22 (0–100)
Premenopausal (R) (*n* = 26) Postmenopausal, (*n* = 56)	0.1 (6.2)	**0.03 (4.9)**	**0.03 (7.3)**	**0.03 (7.3)**	0.5 (1.6)	**0.02 (5.8)**	**0.02 (5.8)**	0.5 (1.7)
T <3 cm (R) (*n* = 59) T>3 cm, (*n* = 23)	0.5 (1.6)	0.18 (0.13)	0.5 (0.29)	0.5 (0.2)	**0.07 (3.2)**	0.3 (2.5)	0.5 (2.5)	0.3 (0.3)
Node negative (R), (*n* = 44) Node positive, (*n* = 38)	0.5 (1.8)	**0.07 (4.3)**	0.35 (2.8)	0.3 (2.2)	0.8 (1.2)	0.1 (0.2)	0.1 (0.2)	0.7 (1.3)
Breast volume medium (R), (*n* = 42) Large, (*n* = 40)	0.09 (3.6)	0.7 (0.7)	**0.07 (5.4)**	**0.07 (7.4)**	0.4 (0.5)	0.7 (1.3)	0.7 (1.3)	0.8 (0.8)
SCF RT (yes) (*n* = 35) SCF RT (No) (*n* = 47)	0.9 (0.9)	**.09 (0.2)**	0.13 (0.17)	0.2 (1.8)	**0.02 (0.1)**	0.9 (1)	0.9 (1)	0.1 (0.3)

Statistically significant which was a p-value of <0.05
